# The prevalence of suicide attempt and suicidal ideation and its relationship with aggression and bullying in Chilean adolescents

**DOI:** 10.3389/fpsyg.2023.1133916

**Published:** 2023-05-18

**Authors:** Constanza Veloso-Besio, Alejandro Cuadra-Peralta, Lorena Gallardo-Peralta, Pascal Cuadra-Fernandez, Pedro Trujillo Quiroz, Nicole Vega Troncoso

**Affiliations:** ^1^University of Tarapacá, Arica, Chile; ^2^Escuela de Psicología y Filosofía, Universidad de Tarapacá, Arica, Chile; ^3^Complutense University of Madrid, Madrid, Spain; ^4^Andres Bello University, Santiago, Santiago Metropolitan Region (RM), Chile

**Keywords:** suicidal ideation, aggression, bullying, suicide attempt, adolescents, suicide prevalence, suicidal behavior

## Abstract

**Background:**

Suicide constitutes one of the main mental health problems worldwide, requiring detection, and prevention efforts, especially in the adolescent population.

**Objective:**

The purpose of this study was to estimate the prevalence of suicide attempts and suicidal ideation and their relationship with aggressiveness and bullying in Chilean adolescents.

**Materials and methods:**

The sample was composed of 728 adolescents schooled from Arica city: 56.6% were males and 43.4% were females. The students attended from de 1st to the 4th year of secondary. The average age of the sample was 15,6 years. The following instruments were used: Okasha’s Suicidality Scale, Buss and Perry’s Aggressiveness Survey, and the Social Acceptance (School Bullying) sub-test of the Kidscreen-52 Survey. A cross-sectional, descriptive, and correlational design was applied. The sampling was non-probabilistic for convenience.

**Results:**

18.4% of the students reported that they had attempted suicide and 65.6% reported that they had suicidal ideation. The prevalence of suicide attempts was higher than in male adolescents (29,1% vs. 10,2%), and the same prevalence was for suicidal ideation (76,6% vs. 57,3%). Suicide attempts and suicidal ideation were positively and significantly correlated with aggressiveness (*r* = 0.32, *r* = 0.48) and bullying (*r* = 0.37, *r* = 0.50).

**Conclusion:**

The prevalence of suicide attempts and suicidal ideation is both higher in girls than boys. In this sense, girls constitute a risk group. In addition, this study provides evidence that supports the relationship between suicide attempts and suicidal ideation. The results highlight the role that educational institutions should have in terms of prevention and effective approaches.

## Introduction

Suicide is one of the most critical public health problems (Glenn et al., 2020) being the second leading cause of death among adolescents aged 15 to 19 years in the United States (Peprah et al., 2023). In more specific terms, Uddin et al. (2019) have pointed out that suicide is the third leading cause of death among male adolescents (10–24 years) and is the most common cause of death among female adolescents (15–19 years). Despite these data, the authors stated that relatively little is known about the epidemiology of adolescent suicide in low- and middle-income countries globally; at the same time, there is little knowledge regarding effective suicide prevention programs.

Regarding suicide in Chile, between 2000 and 2017, the average mortality rates were 8,5 (6,292 deaths), 5,4 (2,676 deaths), and 14,7 (3,616 deaths) per 100,000 in the age groups of 10–24, 10–19, and 20–24, respectively. The national average rate in the 10–24 age group remained at 8,5 per 100,000 between 2000 and 2008 and 2009 and 2017. These data are disturbing because they show that the strategies implemented have not decreased the suicide rate, which makes their approach more urgent to establish preventive measures. Along the same line, the highest number of suicides found in young people between 20–24 years of age reveals the importance of carrying out suicide prevention efforts at an early age (Araneda et al., 2021).

Suicide is the final stage in a continuum of suicidal behaviors. Following Romero et al. (2016), there is a variation in how suicidal behaviors are defined, and they usually include distinctions between suicide ideation (thinking about suicide), suicide plans (making a specific plan for a suicide), and suicide attempts (serious actions of self-harm with the expectation that one would die). Other scholars consider this process also includes consummated suicide, pointing out that suicidality is a continuum of suicidal behaviors that end in suicide achievement (Salvo and Melipillán, 2008; Campillo Serrano and Fajardo Dolci, 2021; Cuadra-Peralta et al., 2021). In the present investigation, both suicidal ideation and suicide attempt will be addressed, since in the prevention of suicide, the role of detecting suicidal ideas and attempts is undeniable (Salvo and Melipillán, 2008).

According to Baiden and Tadeo (2020), various scholars have established that suicidal ideation is one of the most common risk factors associated with suicide attempts and suicide in the future; furthermore, the authors indicate that, according to the evidence available, those who ever seriously considered suicide were four times more likely to have attempted suicide. As stated, suicidal ideation is a risk factor for a suicide attempt, and the latter is the main risk factor for suicide, which constitutes a significant burden for health services due to the treatment of injuries, the psychosocial impact, and the eventual long-term disability (Dávila-Cervantes and Luna-Contreras, 2019).

International findings have shown disturbing figures regarding suicidal ideation and intention (Hong et al., 2016; Romero et al., 2016). The data available to date, from the review by Uddin et al. (2019), which included a total of 229,129 adolescents (13–17 years) from 59 low- and middle-income countries, showed that the general prevalence of suicidal ideation was 17.0% and for suicide attempt was 17.0%. In turn, female adolescents had a higher prevalence of suicidal ideation than male adolescents (18.5% vs. 15.1%); the same prevalence was found for the case of suicide attempts (17.4% vs. 16.3%). Concerning age, adolescents from 15 to 17 years had a higher prevalence of suicidal ideation (17.8% vs. 15.9%) and suicide attempts (17.6% vs. 16.2%) than those from 13 to 14 years, respectively.

In the study by Uddin et al. (2019), it was not possible to find data for Chile (a middle-income country). This situation is alarming since in the region of the Americas the countries with the highest rates of adolescent suicide were Chile, Ecuador, Guyana, Nicaragua, El Salvador, and Suriname (Araneda et al., 2021), which forces us to continue making efforts to make visible the Chilean reality to enrich and deepen future international comparative studies.

Among Chilean studies about suicide attempts and suicidal ideation in the adolescent population is the publication by Ventura-Juncá et al. (2010), who reported a prevalence of suicide attempts of 19% and suicidal ideation of 62%. In another study realized in a sample from Santiago (the capital of Chile), Salvo and Castro (2013) found a prevalence of attempted suicide of 19.1% and the prevalence of severe suicidal ideation was 34.3%. Valdivia et al. (2015) found a prevalence of 16.4% of suicide attempts in a rural district (Concepción City). Silva et al. (2017) reported that 14.3% had intended suicide. Cuadra-Peralta et al. (2021) found 34.5% suicidal ideation in non-consulting young people.

Concerning gender, suicide attempts and suicidal ideations at the national level are more frequent in female adolescents. For example, Salvo and Melipillán (2008), in a study carried out with adolescents from 1st to 4th grade (last 4 years of school education, *n* = 763), reported that female adolescents presented suicidality scores (suicidal ideation and intent) significantly higher than male adolescents and that the effect size of this difference was of medium magnitude. The figures showed that 25.2% of the female adolescents had attempted suicide, compared to 13% of the male adolescents. Regarding age, its relationship with suicidality had low intensity, being the predictor that explained the least of the criterion variable. Ventura-Juncá et al. (2010), in a sample of adolescents, who coursed secondary education (*n* = 1,567, 14–20 years), found a prevalence of 71% suicidal ideation in girls and 26% had attempted suicide. Among the male adolescents, 49% of them had suicidal ideation and 12% had committed suicide attempts. The authors also reported that female adolescents had four times as many attempts as male adolescents. Regarding age, the highest incidence of severe ideation (thoughts of ending life) occurred at 15 years, 1 year earlier than the highest frequency of suicide attempts (16 years). Salvo and Castro (2013), in their study (*n* = 763, 14–19 years), reported that female adolescents presented significantly higher scores on suicidality (suicidal ideation and attempt). Age was not related to suicidality. Valdivia et al. (2015), in a sample of adolescents (*n* = 751, 14–20 years), reported that female adolescents had committed more suicide attempts than male adolescents. Age was not associated with suicide attempts. Silva et al. (2017), in their study of school-aged adolescents (*n* = 919, 13–18 years), also found that females had committed more suicide attempts than male adolescents. Recently, Cuadra-Peralta et al. (2021), in their investigation (*n* = 1,083, 15 average years), reported results that go in the same direction as previous research, i.e., female adolescents had a higher percentage of suicidal ideation (43%) than males (25%).

This research seeks to contribute not only to the study of the prevalence of suicidal ideation and suicidal attempts in adolescents but also to the relationship between some factors that are associated with those components of suicidal behaviors such as aggressiveness and bullying, which require more studies, especially if one considers that these factors can be worked on in educational establishments.

International studies have found an association between suicidal behavior and the preexistence of some psychiatric disorders, mainly with the presence of depression (Turecki and Brent, 2016; Gijzen et al., 2021). In Chile, the association between suicidal ideation with depression, anxiety, stress, emotional dysregulation, and gender in non-consultant school children has been studied. Significant and positive correlations were found between these variables and suicidal behaviors (Cuadra-Peralta et al., 2021). In this line, emotional problems are not treated in schools but referred to the public health system. Variables included in this study such as bullying and aggression correspond to the field of intervention in schools.

Even though bullying harms mental health, this is not a mental health problem but a problem that occurs within the school environment, like most peer aggression (Leff and Waasdorp, 2013), so its solution is the intrinsic responsibility of educational establishments. Thus, although bullying and aggressiveness are not new phenomena (Leff and Waasdorp, 2013), they are of interest to schools, so they should be, although not the only ones, the first and main ones involved, which is consistent with what Puhy et al. (2022) says.

The study of these variables provides meaningful information to give feedback and enrich school convivence programs, which is the task of educational establishments. Adolescents who have suicidal ideation have deep psychological discomfort and, often, it is due to bullying. This discomfort is usually manifested aggressively (fighting, insulting, and getting angry), i.e., they are adolescents who are aggressive in their behavior. However, the actions in response to this are frequently disciplinary and oriented toward punishment such as sending the student to the director, suspending the student for a certain number of days, and calling the parents. Although these measures are part of the actions the school can take, the matter is more complex because the school tends to normalize these behaviors, aggravating their adverse effects.

Aggression and bullying are two concepts that are often used interchangeably (Leff and Waasdorp, 2013). However, various experts on the subject have established their differences. In this sense, aggression between peers is defined as an intentional behavior of a negative or aggressive nature, i.e., directed toward another peer, and bullying is understood as a subset of aggressive behaviors that are systematic, i.e., that are repeated over time and within a context of an imbalance of power between the victim and the perpetrator (e.g., physical and social). For Puhy et al. (2022), every act of bullying is aggression but not all aggression is bullying.

For Buss and Perry (1992), “aggression consists of four subtraits. Physical and verbal aggression, which involves hurting or harming others represent the instrumental or motor component of behavior. Anger, which involves the physiological arousal and preparation for aggression represents the emotional or affective component of behavior. Hostility, which consists of feelings of ill will and injustice represents the cognitive component of behavior” (p. 457). According to the authors, this personality trait can result in threatening or violent behavior. It is worth mentioning that this definition arose from the results of the elaboration of their aggressiveness questionnaire, which was used in this investigation.

Regarding the relationship between aggressiveness and suicidal attempts and suicidal ideation, the findings have provided mixed results. For example, Wang et al. (2014), in a study with a Chinese sample, concluded that students who manifested greater aggression were more susceptible to committing suicide, taking suicide as an expression of aggressiveness. In a recent meta-analysis (*k* = 77), Moore et al. (2022) studied the relationship between aggression and suicidality. The authors reported positive correlations of weak magnitude between aggression and suicidality (*r* = 0, 23). Specifically, the correlation between suicidal ideation and aggression was a weak magnitude and statistically significant (*r* = 0, 24, *p* < 0, 0001, *k* = 38). Regarding the relationship between suicide attempts and aggression, the correlation was weak and not significant *r* = 0, 04, *p* = 0, 033, *k* = 18). The authors also reported that the heterogeneity was significant and only partly explained by moderators, thus suggesting that further studies in the field are needed to explore the relationship between aggression and suicidality. In China, Xuan et al. (2023), in a sample of 2,292 first-year university students, found that physical aggression (*r* = 0, 31), verbal aggression (*r* = 0, 23), anger (*r* = 0, 28), and hostility (*r* = 0, 28) significantly correlated with the suicide risk score.

Bullying is a form of victimization that occurs when a student is attacked or is exposed, repeatedly, and for some time, to negative actions carried out by another student or a group. These actions can be through physical contact, words, or other forms of intimidation. In the dynamics of bullying, various roles can be assumed such as an aggressor, victim, or observer (Smith and Ananiadou, 2003). The present investigation addresses only the role of the victim since the victims are the most vulnerable actors, which is why they are considered a risk group (Rojas Andrade and Leiva Bahamondes, 2015).

At an international level, there is evidence of the negative effect bullying has on different variables of interest. In this line, according to Peprah et al. (2023), bullying is a public health and social issue which harms the development, well-being, and mental health of children and adolescents and makes them susceptible to suicidal behaviors. At the regional level (South America), in Chile, according to the National Youth Institute (INJUV, 2020/2021), various studies have reported that this phenomenon has adverse effects both emotionally and physically on its victims, causing low self-esteem, absenteeism, rejection of school and peers, states of anxiety, depression, increased risk of self-harm, and even suicide. In turn, according to a study carried out on Chilean students (*n* = 1,000, 9–17 years), those who suffered bullying experienced higher rates of distress, especially among peers who knew each other previously (Trajtenberg et al., 2021).

Unfortunately, despite its consequences, bullying victimization is not usually perceived, as it is considered a habitual behavior at school age and only becomes meaningful when its effects have already become severe and even fatal (Rojas Andrade and Leiva Bahamondes, 2015).

Regarding the relationship between bullying, suicidal ideation, and suicide attempts, Holt et al. (2015), in a meta-analytic review (*k* = 41), found a statistically significant average OR of moderate size for bullying victimization and suicidal ideation (OR, 2,34). For bullying victimization and suicidal behaviors (*k* = 18), the results again indicated a significant and moderate average effect size (OR, 2,94). The authors concluded that being a victim of bullying is associated with a high risk of suicidality. Romo and Kelvin (2016) analyzed surveys from five Latin American countries (Chile was not there) to contrast the association between bullying victimization and suicide risk (*n* = 14,560 schooled adolescents). Bullying victimization was associated with higher odds of suicidal ideation (Adjusted Odds Ratio [AOR]: 2,51, *p* < 0,0001), one suicide attempt (AOR: 3,07, *p* < 0, 0001), and multiple attempts (AOR: 4,03, *p* < 0, 0001). In turn, Baiden and Tadeo (2020) found that adolescents who experienced school bullying victimization had 3.67 times higher odds of experiencing suicidal ideation (OR = 3,67, *p* < 0,001). According to Baiden and Tadeo, various researchers have indicated that bullying not only correlates with suicidal ideation but that it is also a predictor of suicidal ideation. Similar findings reported by Galván et al. (2020) showed that adolescents who experience bullying have a higher risk of both suicidal ideation and suicidal behavior. In an international integrative qualitative review (*k* = 18) on the risk factors for suicide and bullying, this was a factor that increased the risk of suicide in adolescents. However, in this review, many studies addressed bullying and suicide separately, but few analyzed the two together (Cuesta et al., 2021).

Finally, in a recent study, Peprah et al. (2023) investigated the association between bullying victimization and suicidal behavior in a sample of 78,558 school-going adolescents (92% under 18 years) from 28 countries. The findings showed that bullying was positively, statistically, and significantly associated with suicidal behavior [relative risk [RR] = 1.44]. Additionally, 23.1% of adolescents experienced bullying, and this variable was associated with a 44% increased risk of suicidal behavior. In another more detailed analysis, the authors contrasted the associations between different forms of bullying victimization (kicked, pushed, or shoved; made fun of because of race; made fun of because of religion; made fun of about sex; left out of activities; made fun of about body; some other way) and subtypes of suicidal behavior (considered suicide, had a suicide plan, or attempted suicide). The results showed that those who had experienced all forms of bullying were at an increased risk of exhibiting all three types of suicidal behavior (*p* < 0,001), compared with those who did not experience any form of bullying.

In light of the above, this research had two general objectives: (1) to estimate the prevalence of suicide attempts and suicidal ideation in the adolescent population of Arica City and (2) to establish the relationship between aggressiveness and bullying with suicide attempts and suicidal ideation in the adolescent population of Arica city.

There are six hypotheses to operationalize the second general objective:

*H1*: *Aggression has a positive and statistically significant correlation with suicidal ideation*.

*H2*: *Aggression has a positive and statistically significant correlation with suicide attempts*.

*H3*: *Bullying has a positive and statistically significant correlation with suicidal ideation*.

*H4*: *Bullying has a positive and statistically significant correlation with suicide attempts*.

*H5*: *Both aggression and bullying have a positive and statistically significant correlation with suicidal ideation in male adolescents and female adolescents*.

*H6*: *Both aggression and bullying have a positive and statistically significant correlation with suicidal attempts in male adolescents and female adolescents*.

## Materials and methods

### Participants

The sample consisted of 728 adolescents who belonged to five free schools in the city of Arica. The total student population of the city corresponded to 13,174 adolescents. Because of the availability and difficulty of access to the sample (e.g., refusal of the schools to participate), the sampling was non-probabilistic for convenience. The inclusion criteria were belonging to either of the two genders, attending secondary school, and belonging to the city of Arica.

The average age of the sample was 15.6 years (SD = 1,27), with a range from 13 to 19 years. Regarding the distribution by gender, 56.6% (*n* = 412) were male adolescents and 43.4% (*n* = 316) were female adolescents. Regarding the distribution of grades (courses), each had approximately the same percentage of adolescents. In the first grade, there were 24.7% (*n* = 180) adolescent students, in the second 28.6% (*n* = 208), in the third grade 25.8% (*n* = 188), and in the fourth grade 20.9% (*n* = 152; See Table 1).

**Table 1:** Characteristics and distribution of the sample according to age, gender, and grade.

Age	15.6 (SD = 1.27)	
Gender		
*Masculine*	412	56.6%
*Feminine*	316	43.4%
Grade		
1st	180	24.7%
2nd	208	28.6%
3rd	188	25.8%
4th	152	20.9%

### Instruments

*Okasha Suicidality Scale* (Okasha et al., 1981) is a 4-item self-administered scale. The first three items explore suicidal ideation (e.g., item 3, “Have you ever thought about ending your life?”), and the fourth item asks about suicide attempts (“Have you tried to commit suicide?”). For each suicidal ideation item, there are four response alternatives: *never, almost never, sometimes, and often*. The response options for suicide attempts are also four: *no attempt, one attempt, two attempts, and three or more attempts*. This scale was validated in the Chilean adolescent population by Salvo et al. (2009), who reported that the suicidality scale showed high internal consistency (*α* = 0.89) and appropriate validity evidence (discriminant and concurrent). The internal consistency for this investigation was 0.87.

*The Buss and Perry Aggression Questionnaire* (AQ, 1992) was studied psychometrically in Chile by Valdivia-Peralta et al. (2014). The questionnaire used in this research was the version of Valdivia-Peralta et al., which is composed of a total of 29 items. The response format is Likert-type with five response options ranging from one (*extremely uncharacteristic of me*) to five (*extremely characteristic of me*). The AQ explores the presence of physical and verbal aggression as well as feelings related to aggression such as anger and hostility. The authors reported that “the AQ scale has appropriate psychometric properties in terms of internal consistency, test–retest reliability, convergent validity, and discriminant validity” (p. 39). However, since there are no records of its use in an equivalent population, the scales were reviewed and refined. According to our review, the reliability for the total scale was *α* = 0.88. The final scales and their reliability, estimated using Cronbach’s alpha, were *α* = 0.83 for physical aggressiveness after eliminating item 29 (item 8, e.g., “If someone hits me, I hit him back”), *α* = 0.69 for verbal aggressiveness (item 5, e.g., “When people annoy me, I argue with them”), *α* = 0.76 for anger (item 7, e.g., “Sometimes I get so annoyed that I feel I’m going to burst), and *α* = 0.72 for hostility after eliminating item 20 (item 7, e.g., “Sometimes I feel that life has treated me unfairly). The two eliminated items had a low item-test correlation.

The KIDSCREEN-52 was used to measure bullying. We apply the Chilean adaptation and validation for children and adolescents carried out by Sepúlveda et al. (2013). The KIDSCREEN measures health-related quality of life and has 10 dimensions. For this research, the Social Acceptance dimension was applied, which explores the feeling of feeling rejected by others as well as the feeling of anxiety toward peers. This dimension has three items (e.g., “Have other girls and boys bullied you?”), whose response format is Likert-type with five options ranging from one (*never*) to five (*always*). The reported reliability was *α* = 0.70. It is worth mentioning that in the original version of KIDSCREEN, created by Ravens-Sieberer et al. (2006), the name given to this dimension was Bullying (see p. 118). In the study by Sepúlveda et al. (2013), the three items of the original version by Ravens-Sieberer et al. were maintained. In the present study, a Cronbach’s alpha coefficient of 0.74 was obtained.

### Procedure

In this research, a cross-sectional, descriptive, and correlational design was applied.

This research was conducted in Chile, which is a middle-income country located in South America. More specifically, the surveys were applied to students from the city of Arica, which is located in the extreme north of Chile, which is quite far from the country, where the nearest city is 300 km away. It has 189,644 inhabitants. In Chile, there are two types of schools, private and non-private (unpaid). The majority of students attend free schools.

Data was collected through a booklet that contained the different questionnaires. The responses were anonymous, and the application modality was self-administered. To apply the questionnaires, authorization was requested from the Director of each educational institution and the teachers, in whose classes the questionnaires were applied. Informed consent was requested from the parents/guardians, in which the conditions of the research, guarantees of confidentiality, anonymity, and contact details of the responsible researchers were communicated. In addition, informed assent was requested from the students, emphasizing that participation was voluntary and anonymous. The application was supervised by the researchers to explain how to answer the booklet and resolve doubts.

### Analytical strategy

The data were analyzed with the statistical program IBM SPSS v.22. Reliability of the scales was estimated by evaluating their internal consistency with Cronbach’s alpha coefficient. To respond to the first general objective of this research (to estimate the prevalence of suicide attempts and suicidal ideation), descriptive analyses were carried out through frequency distribution. To contrast each of the six hypotheses corresponding to the second general objective (to establish the relationship between aggressiveness and bullying with suicidal intent and suicidal ideation), bivariate correlation analysis, t-test for independent samples, and multiple linear regression were carried out. The statistical significance level was 0.05.

## Results

Concerning the first general objective on the prevalence of suicide attempts, the results showed that 18.4% of adolescents indicated that they had attempted suicide at least once. Female adolescents had a higher prevalence of suicide attempts than male adolescents at 29.1% vs. 10.2% (Table 2).

**Table 2:** Suicidal attempts by gender.

Categories	Gender	Quantity	Percentage
No attempts			
	Male adolescents	370	89.8%
	Female adolescents	224	70.9%
	Total	594	81.6%
One or more attempts			
	Male adolescents	42	10.2%
	Female adolescents	92	29.1%
	Total	134	18.4%

The scores for suicide attempts were higher in female adolescents (*x̄*= 1,53; SD = 0,55) than in male adolescents (*x̄*= 1,16; SD = 0,96), and the difference was statistically significant (*t* = 6,12; *p* = 0,000). Even though the statistical contrast of age was not part of the objectives of this research, the analyses were carried out since various investigations (see introduction section) on this variable were addressed. Regarding this variable, there were no statistically significant differences (*x*^2^ = 2,49; gl = 6; *p* = 0,659). The age was operationalized by range/course.

Concerning the prevalence of suicidal ideation (general objective 1), the results revealed that 65.8% of adolescents had presented suicidal ideas, 39.8% had mild suicidal ideas, and 25.8% corresponded to risky (severe) suicidal ideas. Female adolescents had a total prevalence of 76.6% suicidal ideations, 39.9% moderate suicidal ideation, and 36.7% severe suicidal ideation. Male adolescents presented a total prevalence of 57.3%, while the prevalence of severe suicidal ideation was 17.1% (Table 3).

**Table 3:** Suicidal ideation by gender.

Categories	Gender	Quantity	Percentage
No suicidal ideation			
	Male adolescents	176	42.7%
	Female adolescents	64	23.4%
	Total	250	34.3%
Mild suicidal ideation			
	Male adolescents	164	39.8%
	Female adolescents	126	39.9%
	Total	290	39.8%
Severe suicidal ideation			
	Male adolescents	72	17.5%
	Female adolescents	116	36.7%
	Total	188	25.8%

Suicidal ideation scores were higher in female adolescents (*x̄*= 2.18; SD = 1.01) than in male adolescents (*x̄*= 1.7; SD = 0.81), and the difference was statistically significant (*t* = 6.4, *p* = 0.000). Regarding age, there were no statistically significant differences (*x*^2^ = 4.603; gl = 6; *p* = 0.516).

The second general objective had six hypotheses. Regarding H1, *aggression has a positive and statistically significant correlation with suicidal ideation*, a positive correlation of medium magnitude and statistical significance was found (*r* = 0.48, *p* = 0.000). The correlations between the subscales of aggression with suicidal ideation were estimated: physical aggression *r* = 0.32 (*p* = 0.000), verbal aggression *r* = 0.24 (*p* = 0.000), anger *r* = 0.45 (*p* = 0.000), and hostility *r* = 0.56 (*p* = 0.000). As can be seen, all the correlations were positive and statistically significant, with effect sizes ranging from small to large (Table 4).

**Table 4:** Correlations between suicide attempt and suicidal ideation with aggressiveness and bullying.

	Suicidal attempt	Suicide ideation
Total aggressiveness	0.32** (*p* = 0.000)	0.48 (*p* = 0.000)
*Physical aggressiveness*	0.23** (*p* = 0.000)	0.32 (*p* = 0.000)
*Verbal aggressiveness*	0.18** (*p* = 0.000)	0.24 (*p* = 0.000)
*Anger*	0.31** (*p* = 0.000)	0.45 (*p* = 0.000)
*Hostility*	0.32** (*p* = 0.000)	0.56 (*p* = 0.000)
Bullying	0.37** (*p* = 0.000)	0.50 (*p* = 0.000)
Suicide attempt		0.50 (*p* = 0.000)

***p < 0.001.

For H2, *aggression has a positive and statistically significant correlation with suicide attempts*, the correlation was positive, of medium magnitude, and statistically significant (*r* = 0.32, *p* = 0.000). The correlations between the aggression subscales with suicidal intent were contrasted, obtaining the following results: physical aggression *r* = 0.23 (*p* = 0.000), verbal aggression *r* = 0.18 (*p* = 0.000), anger *r* = 0.31 (*p* = 0.000), and hostility *r* = 0.32 (*p* = 0.000). All correlations between the subscales and aggression were positive and statistically significant, with effect sizes ranging from small to medium in magnitude (Table 4).

For H3, *bullying has a positive and statistically significant correlation with suicidal ideation*, the correlation was positive, of moderately strong magnitude, and statistically significant (*r* = 0.50, *p* = 0.000; Table 4).

Regarding H4, *bullying has a positive and statistically significant correlation with suicide attempts*, the correlation was positive, of moderate magnitude, and statistically significant (*r* = 0.37, *p* = 0.000; Table 4).

Additionally, the correlation between suicide attempts with suicidal ideation was analyzed. The correlation was positive and statistically significant (*r* = 0.50, *p* = 0.000; Table 4). Correlations by gender were also made, finding no differences.

To delve into the results, since the independent variables correlated positively and were statistically significant, a multiple regression analysis was performed to determine their joint contribution to suicide attempts and suicidal ideation. Since there was a statistically significant difference by gender, separate analyses were performed for male adolescents and female adolescents.

To respond to the above, H5 and H6 were contrasted (Table 5). Concerning H5, *aggression and bullying together have a positive and statistically significant correlation with suicidal ideation in male adolescents and female adolescents*, for male adolescents, aggression (*β* = 1.36; *p* = 0.000) and bullying (*β* = 1.13; *p* = 0.000) had a multiple correlation of *R* = 0.58, while in female adolescents, the dimensions of aggressiveness (*β* = 1.70; *p* = 0.000) and bullying (*β* = 1.20; *p* = 0.000) had a multiple correlation of *R* = 0.61.

**Table 5:** Regression analysis for the explanation of suicide attempt and suicidal ideation in male adolescents and female adolescents, using aggressiveness and bullying as independent variables.

	Suicide ideation					
Gender		R	F	*P*	*ß*	*P*
Male adolescents	DV suicide attempts	0.579	105.02	0.000		
	Aggressiveness				1.36	0.000
	Bullying				1.13	0.000
Female adolescents	DV suicide attempts	0.607	91.2	0.000		
	Aggressiveness				1.70	0.000
	Bullying				1.20	0.000
	Suicide attempts					
		R	F	*P*	*ß*	*P*
Male adolescents	DV suicide ideation	0.366	31.72	0.000		
	Aggressiveness				0.200	0.000
	Bullying				0.144	0.000
Female adolescents	DV suicide ideation	0.453	40.33	0.000		
	Aggressiveness				0.356	0.000
	Bullying				0.314	0.000

With respect to H6, *aggression and bullying together have a positive and statistically significant correlation with suicidal attempts in male adolescents and female adolescents*, in the case of male adolescents, aggression (*β* = 0.200; *p* = 0.000) and bullying (*β* = 0.144; *p* = 0.000) had a multiple correlation of 0.37, while in female adolescents, aggression (*β* = 0.356; *p* = 0.000) and bullying (*β* = 0.314; *p* = 0.000) had a multiple correlation of *R* = 0.45.

## Discussion

This research had two objectives to study the prevalence of suicide attempts and suicidal ideation in adolescents and analyze the relationship between aggressiveness and bullying with suicidal attempts and suicidal ideation in the adolescent population of Arica City.

About the first objective, the results showed that, at a general level, young people who have had suicide attempts were 18.4%. This result is slightly higher than those found internationally by Uddin et al. (2019), who reported a general prevalence of 17% for suicide attempts. In Chile, our results are in the same direction as those informed by Ventura-Juncá et al. (2010) and Salvo and Castro (2013), who reported a general prevalence of 19 and 19.1%. Valdivia et al. (2015) and Silva et al. (2017) found an overall prevalence of 16.4 and 14.3%, lower than the prevalence reported in this study.

In the case of suicidal ideation, the overall prevalence was 66%. In the international scenario, Uddin et al. (2019) found a general prevalence of 16.9%, which is lower than that found in the present investigation. In the national context, Ventura-Juncá et al. (2010) reported an overall prevalence of 62%. This result is similar to ours. Salvo and Castro (2013) and Cuadra-Peralta et al. (2021) reported an overall prevalence of 34.3 and 34.5%, lower than the prevalence reported in this study.

It is worth mentioning that 26% of adolescents had (severe) risky suicidal ideas, which is in the same direction as that reported by Salvo and Castro (2013), who reported a prevalence of severe suicidal ideation of 34.3%.

In relation to gender, the findings found in this study showed that the prevalence of suicide attempts in female adolescents (29.1%) was higher than in male adolescents (10.2%). At the international level, Uddin et al. (2019) reported that female adolescents had a higher prevalence of attempts than male adolescents (17.4% vs. 16.3%%), but in our study, the prevalence was markedly higher although in the same direction, i.e., there is a higher prevalence in female adolescents. Regarding Chilean research, Salvo and Melipillán (2008) reported a prevalence of suicide attempts of 25.2% for female adolescents and 13.0% for male adolescents. Ventura-Juncá et al. (2010) reported a prevalence of attempts of 26% for female adolescents and 12% for male adolescents. Salvo and Castro (2013) found higher suicidality scores (attempts and ideation) in female adolescents than in male adolescents. Valdivia et al. (2015) found that female adolescents had made more suicide attempts than male adolescents. As can be seen in both the previous evidence and that reported in this research, there is an agreement that female adolescents have a higher suicide attempt than male adolescents.

Regarding the prevalence of suicidal ideation by gender, the findings of this research showed that female adolescents had a prevalence of 76.6%, of which 36% corresponded to severe ideation. Regarding male adolescents, 57.3% have had suicidal ideation in their lives, and 39.8% of them have been severe. At the international level, Uddin et al. (2019) found a higher prevalence in female adolescents than in male adolescents (18.5% vs. 15.1%), but those results were markedly less than the findings of the present investigation. In relation to the national studies, Ventura-Juncá et al. (2010) found a prevalence of 71% for female adolescents and 49% for male adolescents. Cuadra-Peralta et al. (2021) found a prevalence of 43% for female adolescents and 25% for male adolescents. These two national studies generally coincide with the results found in this investigation. As can be seen in both the previous evidence and that presented in this investigation, there is an agreement that female adolescents have higher suicidal ideation than male adolescents.

Regarding the second general objective, about the correlation between suicide attempts and aggressiveness, the results of the present investigation (*r* = 0.32) go in the same direction as that reported by Wang et al. (2014), who concluded that aggressive students had more suicide attempts. However, our findings do not agree with those reported in the meta-analytic review by Moore et al. (2022), who did not find a statistically significant correlation between these two variables (*r* = 0.04). A possible explanation for this discrepancy is the excess of heterogeneity that was reported. In the meta-analysis by Moore et al., the analysis was carried out only over 18 studies, which shows that more research is needed on the subject. Regarding the association between suicide attempts and aggressiveness subtraits (physical aggression *r* = 0.23, verbal aggressiveness *r* = 0.18, anger *r* = 0.31, and hostility *r* = 0.32), our results agree with those reported by Xuan et al. (2023) (physical aggression *r* = 0.31, verbal aggressiveness *r* = 0.23, anger *r* = 0.28, and hostility *r* = 0.28).

Regarding the relationship between aggressiveness and suicidal ideation, the finding of this investigation (*r* = 0.48) was somewhat higher but in the same direction as what was reported by Moore et al. (2022), who reported a correlation of *r* = 0.24. The number of studies meta-analyzed by Moore et al. for the case of suicidal ideation was *k* = 38, so probably, the correlation is more informative and susceptible to comparison. In the case of aggressiveness subtraits, in our research, we found that hostility had a correlation of 0.56 with suicidal ideation, which suggests that this is a subtrait of interest to explore with adolescents who manifest it.

In the case of the bullying variable, it had a positive and statistically significant correlation of *r* = 0.37 with suicidal attempts and *r* = 0.50 with suicidal ideation. These findings go in the same direction as those reported by Holt et al. (2015), who also found associations between bullying victimization and suicidal ideation and suicidal attempt, concluding that being a victim of bullying was associated with a higher risk of suicidality. Romo and Kelvin (2016), Galván et al. (2020), Cuesta et al. (2021), and Peprah et al. (2023) reported similar results to ours. It is worth noting that Baiden and Tadeo (2020) pointed out that bullying not only has a correlation with suicidal ideation but is also a predictor of it. It is worth mentioning that Chile was not included in the Romo and Kelvin study.

Consequently, according to the findings of the present investigation, the relationship between suicide attempt and suicidal ideation with aggressiveness and bullying was positive and statistically significant, i.e., the higher the perception of bullying and aggressiveness, the higher the probability of having suicidal ideation and committing a suicide attempt in adolescents, which goes in the same direction as the previous evidence.

Regarding the results of the regression analysis, it supported the joint contribution of the aggression and bullying variables in the explanation of suicidal ideation, on the one hand, and of suicidal intent, on the other, i.e., both independent variables are potentiated to explain the variance of suicidal behavior in female and male adolescents. Consequently, when both variables are considered, they better predict both suicide attempts and suicidal ideation than either one alone.

The correlation found between bullying and suicidal ideation (*r* = 0.50) is worrisome, as is the correlation found between the hostility subtrait and suicidal ideation (*r* = 0.56) since it provides us with suggestive information about variables that are typical of educational institutions and have adverse impact on suicidal behavior in the adolescent population. Thus, adolescents who are victims of systematic abuse and show aggressive behaviors of hostility (feelings of ill will and injustice) must be cared for and protected as soon as possible due to the potential risk of starting with suicidal ideation. These data reinforce the importance of attending to the first point of the suicidality process, which is suicidal ideation, in the sense that schools should take the presence of this type of idea seriously, exploring its early appearance and activating action and prevention protocols to safeguard the integrity of adolescents. This is not minor since according to Baiden and Tadeo (2020), those who seriously considered committing suicide were four times more likely to do so.

In this sense, bullying and hostility constitute one of the edges that schools must address in time to reduce suicide attempts and suicidal ideation. This is in line with the findings by Cuesta et al. (2021), who pointed out that early detection and effective action are essential to avoid prolonged situations of bullying, which can have negative consequences such as suicidal ideation, suicidal attempts, or worst of all, the consummation of suicide. This is of particular interest if one takes into account that suicidal ideation is a risk factor for a suicide attempt and the latter is considered the main risk factor for suicide.

### Limitations

The limitation of this study was the use of a cross-sectional design, which reports information on a phenomenon at a specific moment, so it is not possible to follow up on cases of suicidal behavior, especially the most serious ones. Unfortunately, it is difficult to overcome this limitation since the responses are anonymous and the topic is sensitive. This limitation is frequently transversal to this type of study.

### Contributions

One of the contributions of this research is to provide an overview of suicide attempts and suicidal ideation in the non-consulting population and, in addition, the detection of actors that could influence suicidal behavior.

This investigation provides statistics on the prevalence of suicidal ideation and suicide attempts in the north of Chile. These data may be helpful for future international comparative research. In the study by Uddin et al. (2019) on adolescent suicidal behavior in low- and middle-income countries, Chile was not there, which is a middle-income country. The same occurred with the study by Romo and Kelvin (2016) about the association between bullying victimization and suicidal ideation and suicide attempt in which Chile was not included. Consequently, it is necessary to continue developing this line of research to provide specific information to the scientific community about this global problem and make visible the national reality of this middle-income country.

This research also contributes to our country since it provides data on the prevalence of suicidal ideation and suicide attempts in the extreme north of Chile, complementing the data obtained in the central and southern areas of the country. So, the present study joins a line of national research on suicidal behavior. It is very relevant to mention the fact that, both in the reviewed literature and in the results found in this study, a higher prevalence of suicidal ideation and suicide attempt was observed in female adolescents than in male adolescents, so educational institutions should pay attention to this risk group, formulating intervention strategies specially adapted for female adolescents.

Another interesting contribution is that aggressiveness and bullying together explain more variance than each one separately. It is important to emphasize that this research considers both variables together, unlike most previous studies.

As a last contribution, the results of this research provide important information to give feedback and enrich school convivence programs aimed at addressing bullying. As stated in the introduction, adolescents who have suicidal ideation have deep psychological distress and, often, it is due to bullying. These adolescents tend to express this discomfort aggressively, which constitutes a positive reinforcement for the aggressor and a punishment from the school for the adolescent who manifests his suffering aggressively, who is nothing more than a victim. Unfortunately, the victim goes through a double process of victimization, deepening and aggravating the adverse effects of bullying such as suicide attempts and suicidal ideation (Holt et al., 2015; Romo and Kelvin, 2016; Baiden and Tadeo, 2020 INJUV, 2020/2021). Consequently, intervention programs for bullying should take these subtleties to increase their effectiveness.

### Practical recommendations

This study suggests taking preventive measures, focusing interventions on education and awareness of the educational community regarding the problem of suicide, emphasizing the need for adequate training of professionals who have direct contact with adolescents. Also, the detection of aggressiveness and bullying allows a generation of intervention strategies that aim to mitigate suicidal behavior among young people. Aggressiveness and, especially, the proper management and control of anger and hostility, along with bullying, are related to suicidal behaviors, therefore they are topics to consider in the implementation of preventive interventions.

Educational establishments should be attentive to the school climate through systematic interventions to improve and maintain a healthy school climate. This challenge requires a joint effort from all the adults who work in the school. It goes in the same direction as indicated by Cao et al. (2022), who consider that the perceived school climate could moderate the relationship between bullying victimization and suicidal ideation. Along these lines, the authors suggest that a positive school climate could play a protective role in preventing violence, especially bullying victimization.

The classroom climate is another edge that could diminish the suicidal behavior that requires high commitment and patience from the teachers (who have daily and direct contact with students) since their work has broadened from academic formation to contributing to the development of emotional competencies in students and their emotional contention. An adequate classroom climate could impact adolescents´ well-being (Cuadra-Peralta et al., 2012).

At an individual level, adults who work in schools (e.g., teachers, inspectors, yard supervisors, school climate officers, and psychologists) could help the student by establishing a supportive and empathetic emotional bond, who can contain and help them and, above all, who is willing to work with the different parties involved (Leff and Waasdorp, 2013).

We hope this study serves to help health professionals, students, parents, and teachers with the importance of the subject of suicide. More than half of the studied adolescents report suicidal ideations, with a higher prevalence in female adolescents, which provides relevant information to ask what is happening with young people and who is taking care of this possible suffering.

### Suggestions for future research

Future research should focus on the relationship between bullying and suicidal behavior in the foreign population that migrates to Chile since today the country has a massive migratory wave (mainly Venezuelan and Colombian), as stated in a recent newspaper article (Cerda and Davied, 2023), which reported that “the northern macro-zone experiences an overdemand for school enrollment due to the constant increase in foreigners” (p. 11). Arica belongs to the northern macrozone which has been the most affected city by the migratory wave because it shares an adjoining border with Peru and Bolivia, which are the countries through which the migrants enter, either regularly or undocumented (through clandestine routes).

Specifically, it would be convenient to study these variables in Afro-descendant migrants since, until very recently, Chile did not have this population of school age. This recommendation is especially relevant in light of a recent article by Peprah et al. (2023), in which they found that teasing associated with race (a type of bullying) was the second variable out of seven that significantly predicted suicidal behavior. In more detailed terms, it was the third most important predictor associated with considering committing suicide, and it was the second most important predictor of planning and attempting suicide. These reasons suggest that there is a gap in this issue, which is of particular urgency.

### Conclusion

In conclusion, this research provides evidence of the prevalence of suicidal ideation and suicide attempt, both of which are higher in female adolescents than in male adolescents. In this sense, female adolescents constitute a risk group that requires special attention. In addition, this study provides information that supports the relationship between suicide attempts and suicidal ideation with aggression and bullying. The latter are phenomena that occur mainly in the school context and severely affect our adolescents by increasing the probability of the appearance of suicidal ideation and committing suicide attempts. In this line, these results highlight the role that educational institutions should have in terms of prevention and effective approaches.

## Data availability statement

The original contributions presented in the study are included in the article/supplementary material, further inquiries can be directed to the corresponding author.

## Ethics statement

The studies involving human participants were reviewed and approved by CEC UTA. Written informed consent to participate in this study was provided by the participants’ legal guardian/next of kin.

## Author contributions

CV-B and AC-P: research idea, design, data analysis, and writing of the manuscript. LG-P: data analysis support, English editing, and review of manuscript. PC-F: English translation and review of manuscript. PQ and NT: instruments application and database and writing first version of manuscript. All authors contributed to the article and approved the submitted version.

## Funding

This research was supported for PROYECTO DE INVESTIGACION UTA-MAYOR 3773-22.

## Conflict of interest

The authors declare that the research was conducted in the absence of any commercial or financial relationships that could be construed as a potential conflict of interest.

## Publisher’s note

All claims expressed in this article are solely those of the authors and do not necessarily represent those of their affiliated organizations, or those of the publisher, the editors and the reviewers. Any product that may be evaluated in this article, or claim that may be made by its manufacturer, is not guaranteed or endorsed by the publisher.
